# Contributory Schemes at Work

**Published:** 1923-10

**Authors:** 


### CONTRIBUTORY SCHEMES AT WORK.

### VOLUNTARY HOSPITALS COMMISSION REPORT.

At the request of the Conference of representatives of Local Voluntary Hospital Committees held on July 18, 1922, the Commission undertook to issue a questionnaire on the methods pursued in various areas to obtain contributions, and to circulate to the local committees a summary of the information received. A questionnaire was accordingly issued, and particulars were received of 73 contributory schemes, affecting 102 hospitals. A summary of these particulars has now been issued by the Voluntary Hospitals Commission through the Ministry of Health (Stationery Office, 3d. net). It forms a highly interesting and instructive document.

### The Machinery of Contributory Schemes.

The report shows that there is a wide variety in the methods that have been adopted for working contributory schemes :—

At one extreme there are several instances of a single hospital working a scheme through its own secretary and treasurer; at the other, there is a scheme worked by a highly organised hospital council on which there are representatives of all the hospitals in the area, the City Corporation and various trade associations and public bodies. Between these two extremes many varieties of organisation will be found. Some individual hospitals have found it convenient to manage their schemes through a special sub-commiteee; others work in co-operation with the Hospital Sunday Fund and Hospital Saturday Fund. Some, again, have secured assistance from committees based on local areas, such as town wards and country parishes, and in one large rural scheme the work is done chiefly through collectors in each village. On the other hand, in one industrial centre the contributory scheme is worked for the hospital by a Workmen's Hospital Committee composed of representatives from each of the various trades. In other areas of an industrial character the hospitals have found it convenient to work their schemes through Special Aid Committees formed within the various works and trade associations. A number of cases have been noted where two or more hospitals have combined in organising a contributory scheme.

### Methods of Collection.

Almost every scheme relies mainly upon deductions from wages; but there are many other avenues through which funds are collected, and many schemes use them concurrently with deductions :—

For example, in industrial establishments the contributions may be secured by deduction from wages, while the residential population is reached by a systematic house-to-house collection or by periodic applications through the post. In some instances funds are collected through trade associations and other societies. It should be mentioned that valuable help is derived in many areas through the active co-operation of trade unions and employers' associations. The method of collection in a rural area presents considerable difficulty. Two cases have been brought to the Commission's notice in which the areas are partly rural and partly industrial, and in these instances a twofold contributory scheme has been put into operation. For the industrial parts the collection is made by deduction from wages, but in the rural parts a special arrangement is made for the farmers. In one case the contributions are forwarded through the post, the farmers collecting a weekly contribution from the farm hands and adding a similar sum themselves; in another, the farmers have been induced to authorise their bankers to make periodic payments and the district farmers' association has co-operated to make the scheme workable. Mention should be made of one purely rural scheme in which the area is worked through group and village committees and the contributions collected by a voluntary collector in every village.

### Privileges for Contributors.

A large number of schemes have fixed 2d. a week as the sum payable by each contributor. More elasticity, however, is shown in a number of cases, and the amount varies from 1d. a week to 3d. Sometimes it varies at the choice of the contributor, sometimes the larger contribution covers privileges for the employee and his family, while the minimum figure would cover only the employee himself. Most schemes fixed 1d. a week as the minimum; in very few does the contribution exceed 3d. a week :—

The inducement to be held out in order to attract contributors is perhaps the most important and the most difficult feature in framing a contributory scheme. Many hospitals feel the necessity of offering some special inducement in return for the assured income obtained through regular contributions. On the other hand, it is generally recognised in the voluntary hospitals, and indeed often required as a result of trusts and endowments, that the first claim on their accommodation and resources is that of the necessitous poor. Moreover, the governing condition in the admission of patients to a hospital should obviously be the urgency of the medical or surgical need of the case. These considerations have no doubt weighed with the majority of hospitals, who have been careful to avoid holding out any inducement that would bind them in a contractual obligation. It is noteworthy that there are very few instances in which anything approaching an insurance scheme has been attempted, but in one or two such cases the hospital authorities have guaranteed to all their contributors certain definite benefits. It will be appreciated that the adoption of an insurance scheme of this nature in regard to a large number of contributors might involve the hospital authorities in contractual obligations which they might find it exceedingly difficult to discharge.

### Tickets of Recommendation.

A very common form of inducement is the simple practice whereby contributors when admitted to the hospital are maintained and treated free of charge—they are not asked, as are other patients, by the almoners to make a donation to the hospital. Nine or ten schemes have this feature in common, and it is noteworthy that many of them extend this privilege to the contributor, his wife and children under 16. In two cases the hospitals maintain and treat the contributor free of charge and make a reduced charge in the case of dependents :—

A considerable group of schemes offer a different kind of inducement which it may be noted does not confer a special privilege. In these cases the contributors have no difficulty in getting tickets or recommendations for admission. In many cases the contributors obtain recommendations strictly according to the scale of contribution. At least a dozen contributory schemes report that this is the sole inducement on which they work. In two instances the hospital authorities have reported that contributors are given free maintenance when necessary, but that the rules governing priority of admission are the same for contributors as for all other cases. In eight cases promoters of contributory schemes have stated that no inducement or privilege is accorded to the contributors. The hospital authorities rely (and rely successfully) on the charitable instinct of their contributors. The sole criterion of admission is the urgency of the case, and the policy of the open door is rigidly maintained. . . . It is noteworthy that of all the schemes examined only four have indicated that payments by patients have diminished as a result of the operation of a contributory scheme. Many indicate that the one has had no effect on the other; but in a few instances the hospitals anticipate that as the number of regular contributors increase there will of necessity be a diminution in the extent of patients' payments.

### Representation of Contributors.

The representation on the Governing Body of hospitals of the workpeople contributing is obviously important, and there are very few schemes which do not make some provision for such representation:—

The basis on which the contributors are represented may be divided into three principal varieties. Representation in a large number of cases is accorded upon a purely financial basis. Many schemes provide that the contribution of a certain sum (varying from a guinea to £100) shall entitle the contributor to be a governor. The following illustrations may be noted:—In one case firms appoint representatives to a special committee which elects 15 governors of the hospital, who in turn serve on the management committee and house committee. In another case one governor can be appointed by the contributors for every £50 contributed, and they elect one representative on the board of management annually. In another case every firm collecting £5 and upwards is entitled to send a delegate to the annual meeting, which elects five members to the board of management. Another scheme provides that one governor can be elected for each £100 subscribed, and one representative on the board of management for each £1,500 subscribed. In another case employees of every factory who contribute £200 per annum nominate one representative for election to the committee of management. In another instance each workshop or trade union is entitled to appoint one governor for every 10 guineas subscribed annually, and these governors elect from their own number eight members for the board of management. Another basis which appears to be as popular as the financial basis is that contributors as a body are entitled to a fixed number of representatives on the boards of management or governing bodies. The third category consists of cases in which contributors are accorded representation, but not on any fixed basis.

